# Connection and happiness: an interactive study of social media, social capital, and the subjective well-being of international students

**DOI:** 10.3389/fpsyg.2025.1548663

**Published:** 2025-05-09

**Authors:** Ninggui Duan, Hong Lu, Huihui Lyu

**Affiliations:** ^1^School of Humanities and Management, Youjiang Medical University for Nationalities, Baise, China; ^2^School of Language and Culture, Youjiang Medical University for Nationalities, Baise, China; ^3^School of Business Administration, Baise University, Baise, China

**Keywords:** social media, subjective well-being, social capital, college attachment, international student

## Abstract

The subjective well-being of international students is crucial for their adaptation and academic success in a foreign country. Based on online social capital theory, this study examines how social media use affects the subjective well-being of international students and explores the underlying mechanisms and the moderating role of college attachment. A questionnaire survey was conducted among 21 universities in China, yielding 474 valid responses, which were analyzed using structural equation modeling. The findings indicate that social media use significantly and positively predicts the subjective well-being of international students, as well as bridging, bonding, and maintaining social capital. Notably, only bonding social capital partially mediates the relationship between social media use and subjective well-being. Additionally, college attachment positively moderates the indirect effect of bridging social capital only, but it did not significantly moderate the relationship between social media use and subjective well-being. These results highlight that social media use can enhance the subjective well-being of international students through social capital. This effect is moderated by college attachment, providing valuable insights for relevant stakeholders.

## Introduction

Today’s university students, as “digital natives” raised in the information age, are among the most active social media users (Janschitz and Penker, 2022). An increasing number pursue higher education abroad to gain cross-cultural competence and a global perspective (Ng et al., 2017). However, international students often encounter cross-cultural adaptation challenges, which can impede their progress if not effectively addressed (Bukhari et al., 2020). Typically, they rely on social networks for support (Pang, 2018d), utilizing social media to build connections within the host country and relying on it for emotional support and maintaining ties within their home country’s social network (Gao et al., 2021).

Research shows social media use significantly impacts college students, influencing knowledge exchange (Duan and Li, 2023), social capital acquisition (Zhang et al., 2023), information seeking (Guo et al., 2014), uncertainty reduction (Gambo and Ozad, 2021), and ethnic identity. Notably, it correlates with well-being by enhancing individual happiness and life satisfaction (Núñez-Naranjo et al., 2024; Pang and Wang, 2020). Conversely, social media use is also linked to adverse health outcomes, such as addiction (Jiang et al., 2018), fatigue (Zheng and Ling, 2021), privacy violations (Wang et al., 2021), and cyberbullying (Kerapetse, 2018), which can lead to poor academic performance and mental health issues (Duan et al., 2024). Some students isolate themselves within familiar social circles online, avoiding offline interactions in the host country (Yang, 2018). Weak university attachment can also reduce their willingness to communicate, leading to depression, lower subjective well-being, and failed cross-cultural adaptation (Zhou and Zhang, 2019). Thus, social media can be a double-edged sword (Ni and Shao, 2019), influencing physical and mental health, and its complex relationship with individual well-being requires further exploration (Erfani and Abedin, 2018; Pornsakulvanich, 2017).

Social media has become integral to international students’ daily lives, playing a key role in cross-cultural communication (Gao, 2022). However, surprisingly, few studies have systematically examined how social media influences the cross-cultural adaptation of these sojourners (Pang and Wang, 2020). To address this gap, this study explores two questions: “Does social media use affect the subjective well-being of international students?” and “What role do social capital and university attachment play in this relationship?” Given that some social media platforms like Facebook and Instagram are not accessible in China, while others like WeChat and QQ are widely used, this study focuses on instant messaging social media, including WeChat, WhatsApp, and QQ.

## Literature review and theory development

### Online social capital theory

Social capital refers to the structure of resources and benefits potentially accumulated through relationships with others, such as emotional support, helpful information, or economic assistance. Generally, social capital resources are tangible or intangible, encompassing trust, norms, and social networks (Boyd and Ellison, 2007; Ellison et al., 2007). Individuals utilize social media to maintain their existing social networks and forge new social connections based on shared interests, viewpoints, or activities through online communication, thereby gaining social capital (Raza et al., 2020). Building on previous research, Ellison et al. (2007) distinguished three types of online social capital: bridging, bonding, and maintaining social capital.

Specifically, bridging social capital refers to loosening relationships (i.e., weak relationships), reflecting the weak network and loose connections between individuals, and providing relevant information support or functional perspectives for each other (Jahan et al., 2024). Bonding social capital offers social and emotional support through strong connections, emphasizing intimate and trusting relationships such as family members, relatives, and close friends (Zhang and Liu, 2018). Maintaining social capital refers to keeping connections valuable (i.e., past connections) even after life changes. For example, international students in a foreign environment need to establish new circles of friends in the host country and maintain connections with old social networks in their home country (Pang, 2018d). A study based on social capital theory found a positive correlation between Instagram usage patterns and social capital, with only the bonding social capital and life satisfaction being positively correlated and having mediating effects, while bridging social capital was not significant (Reimann et al., 2023).

International students using social media can gain certain social capital and affect their subjective well-being. In an alien cultural environment, international students become a vulnerable group (Nailevna, 2017). Social capital can provide some positive and beneficial resources that can positively impact the lives of international students, especially those with low life satisfaction, enabling them to join and seek social interactions and increase their happiness (Hendrickson and Rosen, 2017). If international students can use social media to maintain and grow social capital, increase trust, and minimize interference with academic activities, positive results can be achieved (Wang et al., 2021). However, those who have more contact with local and other international students report better cross-cultural adaptation. In comparison, those with more contact with their home country tend to exhibit poorer cross-cultural adaptation (Billedo et al., 2019). Additionally, although bridging social capital can expand individuals’ social horizons and information extraction, it does not provide much emotional support to individuals (An and Chen, 2017).

### Relationship between social media, social capital, and subjective well-being

Social media can help international students adapt to the local living environment and enhance their proficiency in the host country’s language. According to statistics, 90% of international students have joined different online virtual communities, mainly consisting of classmates or friends who share the same native language (Sun and Tian, 2020). The use of social media seems crucial for cultivating international students’ cultural adaptation experience and coping strategies, as it provides opportunities to develop language skills and offers more chances to understand the host country’s culture (Hofhuis et al., 2019). Individuals who may face difficulties in face-to-face communication due to shyness or language barriers often experience less pressure on social media (Kim et al., 2020). Moreover, international students are susceptible to the pressure of classmates and teachers (Hwang et al., 2018). After discovering that their Chinese classmates are all using WeChat or QQ, they may exhibit a convergence in media usage (Shang et al., 2021). Therefore, they are willing to accept mainstream Chinese social media and encourage their friends and family in their home country to use these platforms (Kuang and Wu, 2019).

Social media usage contributes to social capital formation through interactions between individuals. Facebook incorporates features that assist users in establishing and maintaining numerous weak ties, thereby enhancing subjective well-being (Verduyn et al., 2017). Social media can foster social connections among international students, improve international learning experiences, and strengthen community awareness (Sleeman et al., 2020; Xiao and Hu, 2021). Social media can eliminate information asymmetry, acquire social capital, and cultivate a sense of identity with the host country’s culture (Billedo et al., 2019). Research has also found that excessive interaction with compatriots may increase psychological barriers, undermining subjective well-being (Hofhuis et al., 2019). When the number of international students and host country students becoming friends is low, their cross-cultural level is still not high, resulting in lower subjective well-being (Gao, 2022).

Social network relationships affect an individual’s subjective well-being. As social animals, humans need to connect with others to thrive, and these social media platforms help people fulfill this basic need (Clark et al., 2018). Similarly, friendship is an essential component of satisfying emotional needs, belonging to Maslow’s third level of needs, which includes the need for belonging and love (Guo and He, 2017). If international students’ cross-cultural adaptation barriers are not addressed for a long time, it will affect their mental health (Li, 2022). International students are prone to cognitive problems and other forms of distress in unfamiliar social contexts, with depression being one of the main psychological issues (Bukhari et al., 2020). Good communication and social support can alleviate depressive symptoms and enhance well-being (Billedo et al., 2019; Ng et al., 2017).

Social capital is essential in the relationship between international students’ social media use and subjective well-being. Studies have shown that the number of social media friends has a significant positive impact on reducing cross-cultural adaptation stress, subjective well-being, and life satisfaction (Forbush and Foucault-Welles, 2016). The use of social media allows international students to exchange information in a networked environment, acquire and develop personal online social capital, promote learning and participation in life, and enhance satisfaction with university life (Kerapetse, 2018; Pang and Wang, 2020). Furthermore, increasing evidence suggests that personality is related to social network behavior, and communication usage and self-disclosure through mobile social media are significantly positively correlated with bridging and bonding capital and well-being outcomes (Chen and Li, 2017; Valkenburg et al., 2022). For example, Wang et al. (2014) also confirmed that the “social” forms of social media use, such as status updates, are significantly positively correlated with users’ subjective well-being. Research on Chinese students studying in the UK has shown that social media allows them to maintain communication with people from their hometowns, alleviating their cultural adaptation stress and enhancing their satisfaction (Yu et al., 2019). Some scholars believe that the connection between social network usage and its social and psychological consequences may depend on the indirect functions of social network usage (Guo et al., 2014). Social media may improve users’ subjective well-being through these key mediators (such as bridging or bonding social capital) rather than directly impacting well-being (Lin et al., 2022; Pang, 2018a). Therefore, social capital may be an intermediary variable in social media use and subjective well-being.

Additionally, college attachment may mediate the relationship between international students’ social media use and subjective well-being. Research has shown that social media use positively correlates with college students’ sense of belonging and satisfaction with campus life (Park et al., 2014). Therefore, international students’ attitudes and perceptions toward their universities can affect their willingness to interact socially (Lu and Duan, 2021). Researchers in the field of social psychology have recognized the unique role of college attachment, which may moderate the impact of different cultural differences and personality traits on international students’ subjective well-being during social media use.

### Hypotheses statement and theoretical model

Social connections or friendships are crucial to a person’s happiness, and numerous studies have empirically demonstrated that social media use positively impacts happiness (Xu et al., 2021; Yoo and Jeong, 2017). Ellison et al. confirmed the positive relationship between the perceived social capital of international students and their satisfaction with campus life (Ellison et al., 2007). Du and Lin (2019) found that the higher the frequency of using social media in the host country, the stronger the sense of cultural identity and the more pronounced the subjective well-being. Gao (2022) also found that the density and type of social network relationships of international students in the host country positively correlate with cultural identity, affecting their happiness. International students use Facebook to establish groups as an example of social media applications to seek information about learning and life, and the results show a positive correlation with happiness (Bukhari et al., 2020). Therefore, we propose hypothesis 1.

*H1:* Social media use positively predicts the subjective well-being of international students

Social media has the potential to reshape social networks and reduce the cost of communication within them. Social media use may significantly impact individuals’ social capital, and its intensity of use is significantly positively correlated with social capital (Johnston et al., 2013). For international students, bridging social capital is particularly important, as it mainly extracts resources and value from weak-tie social networks composed of classmates, colleagues, and strangers, especially in obtaining novel, new, and heterogeneous information (Xu et al., 2021). Some users who fear isolation actively use social media for self-presentation and interaction, thereby gaining social support (Lee and Cho, 2018; Wang et al., 2018). The use of social media by international students is significantly positively correlated with perceived social capital, as it can expand the size of social circles, promote social interaction, and ultimately contribute to the development and maintenance of social capital (An and Chen, 2017; Kuang and Wu, 2019). Through social media, individuals can also rebuild weak connections by engaging with a broader range of users with specific backgrounds. For example, interpersonal communication through digital media technology allows people to overcome time and space barriers at an unprecedented level, communicating with different people who share common interests and needs (Park and Noh, 2018). Jin and Zhang (2017) found that the use of WeChat has a significant positive effect on enhancing Chinese social identity and strong and weak social relationships among international students in China. Therefore, this article proposes the following hypothesis:

*H2:* Social media use positively predicts the bridging social capital of international students

*H3:* Social media use positively predicts the bonding social capital of international students

*H4:* Social media use positively predicts the maintenance of the Social Capital of international students

Those with weak social relationships or feeling isolated can utilize social media as a means of fostering social interactions to meet their needs (Lee and Cho, 2018; Wang et al., 2018). As college students increasingly focus on gaining social capital through these social media platforms, those with lower life satisfaction seek to participate in online social networks to enhance personal happiness (Guo et al., 2018). One study indicates that social media enables individuals to maintain contact with old friends and preserve offline relationships, especially with close acquaintances (Ellison et al., 2007). Those who leverage their social connections when needed will have stronger social ties, and the more social support a person receives, the happier and healthier they are likely to be and the more likely they are to live longer (Dos Santos et al., 2023). Being abroad, some individuals still seek ways to maintain contact with past classmates, and social media serves as a complementary method to strengthen face-to-face social connections, maintaining and enhancing existing social ties (Pang, 2018a). Members of social networks can interact with each other more frequently, and remote relationships can also be managed. Social capital gained through social connections with old neighbors, high school classmates, and social clubs is also likely to correlate positively with one’s mental health and life satisfaction (Pang, 2018c). Based on this, we propose hypotheses H5–H7.

*H5:* Bridging social capital positively predicts the subjective well-being of international students

*H6:* Bonding social capital positively predicts the subjective well-being of international students

*H7:* Maintaining Social Capital positively predicts the subjective well-being of international students

In addition to the direct impact relationships proposed in the hypothesis above, previous studies have also mentioned the mediating role of social capital (Ellison et al., 2007; Pang, 2018b). In this study, social capital is a mediating variable in the relationship between social media use and subjective well-being, focusing on its underlying mechanisms (Erfani and Abedin, 2018). Social capital based on networks and social relationships with others is a key factor in the positive impact of social media use on life satisfaction (Yoo and Jeong, 2017). Individuals’ social media use to interact with friends and groups can improve their psychological well-being (Du and Lin, 2019). So far, only a few studies have revealed whether perceived social capital mediates between social media use and subjective well-being (Kim and Kim, 2017). A recent study found that bonding social capital plays a positive mediating role between active Instagram use and individual life satisfaction (Reimann et al., 2023). Therefore, this article proposes the following hypothesis:

*H8:* Bridging social capital plays a mediating role in the relationship between international students’ social media use and subjective well-being.

*H9:* Bonding social capital plays a mediating role in the relationship between international students' social media use and subjective well-being.

*H10:* Maintaining Social Capital plays a mediating role in the relationship between international students' social media use and subjective well-being.

**Figure 1:**
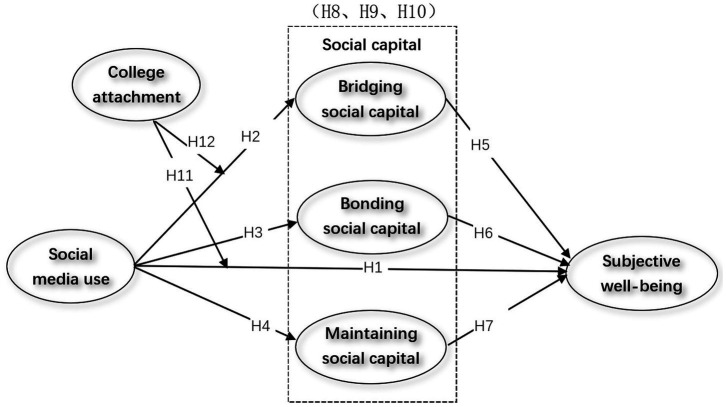
Research model.

Attachment is one of the factors influencing subjective well-being. Multiple studies have shown a positive correlation between students’ attachment to universities and subjective well-being (Kim et al., 2020; Kim et al., 2017). Due to varying environments, personal attachment to universities plays a significant role in daily life, identity recognition, and well-being (Hu and Chen, 2017). Li (2015) found substantial differences in the overall well-being levels of college students with different attachment types. Other scholars have discovered that college students with lower levels of distress experience higher levels of subjective well-being. At the same time, attachment anxiety and avoidance are negatively correlated with subjective well-being (Luo, 2021). Furthermore, attachment to universities can also influence social media use. For instance, an individual’s positive experience with their university can influence their more active use of social media to interact with classmates, suggesting that college attachment may play a moderating role (Pang, 2018c). Considering that international students’ use of social media and interactions with faculty and students at their host university are influenced by their attachment to the university, this affects the acquisition of bridging social capital (Pang, 2018d). Therefore, we propose the following hypothesis and form the research model presented in Figure 1.

*H11:* College attachment plays a moderating role in the relationship between international students’ social media use and subjective well-being.

*H12:* College attachment plays a moderating role in the relationship between international students’ social media use and bridging social capital.

## Materials and methods

The social media use scale adopted the scale developed by Petersen and Johnston (2015), consisting of 6 items, with a sample item as “social media is part of my everyday activity.” The Cronbach’s alpha value of the scale in this study is 0.811. The social capital scale also utilized the scale developed by Petersen and Johnston (2015), which includes three subscales: bridging, bonding, and maintaining social capital scale, totaling 19 items. A sample item is “At my university, I came in contact with new people all the time.” Cronbach’s alpha values are 0.881, 0.871, and 0.919, respectively. The college attachment scale primarily referenced the scale developed by Kim et al. (2017), consisting of 3 items, with a sample item as “I am pleased about attending this university”; Cronbach’s alpha value is 0.826. The subjective well-being scale employed the Psychological Well-Being Scale developed by Diener et al. (2009) to measure individuals’ subjective well-being, consisting of 6 items, with a sample item as “My social relationships are supportive and rewarding”; Cronbach’s alpha value is 0.884. Based on the abovementioned scales, the entire survey questionnaire comprised 34 items, utilizing a Likert 7-point scoring system, ranging from 1 (strongly disagree) to 7 (strongly agree). Detecting the relationship between scales is based on the mean value of each scale. Additionally, there were eight demographic items (including gender, age, nationality, discipline, educational background, social media use history, number of friends, and usage duration).

This study also conducted a pre-survey involving over 50 international students who participated in a pilot test. The results indicated that two items (BR4 and BR8) of the bridging social capital in the social capital scale and one item (SW6) of the subjective well-being scale had excessively low factor loadings and were subsequently deleted, leading to the formation of the formal survey questionnaire. Subsequently, this study surveyed international students from 21 universities in Guangxi, China. Participants were recruited primarily through a combination of stratified sampling and snowball sampling methods. According to Guangxi Daily, in December 2024, the total number of international students studying in Guangxi was approximately 9,000. Initially, based on Zikmund (2003)‘s sample size calculation formula, the minimum sample size was 369. Considering that the snowball method may lead to significant sample bias, the standard of 5–10 times the scale items is usually not applicable to this study. Therefore, we have decided to increase the sample size to 20 times (i.e., 680 samples), first, determine the minimum number of questionnaires distributed by each university using the stratified sampling method, and then achieve sufficient online questionnaire filling through participant diffusion. The survey was conducted from November 9th to November 25th, 2024, with 700 questionnaires distributed and 697 questionnaires collected, resulting in a response rate of 99.5%. The sample data was imported into SPSS for validity testing, and after removing some invalid samples, 474 valid samples were obtained. Finally, we used SPSS 24.0 and Amos 24.0 to conduct reliability and validity tests, correlation analysis, factor analysis, variance analysis, path analysis, and model fit testing using structural equation modeling. Mediating and moderating effects were also tested later.

### Data analysis and results

#### Demographic analysis

From the demographic information, the male-to-female ratio reflects the current situation of male and female international students in Guangxi universities, with significantly more female students than male students. 98.5% of the students are aged 18–25. The number of students in science, engineering, humanities, and social sciences is the same. Most of the students in the sample are undergraduates (65.4%), and the number of students at other educational levels is relatively small. Most students have been using social media for over 3 years (86.5%), indicating that international students have widely used social media. The proportion of students with over 100 friends on social media reaches 87.1%, suggesting that they have many online friends. As many as 80.4% of international students use social media for more than 2 h a day on average, and nearly half of them use it for more than 4 h, indicating that the phenomenon of excessive use of social media is widespread (Table 1).

**Table 1:** Distribution of samples (*N* = 474).

Variable	Option	Frequency	Percentage
Gender	Male	170	35.9
	Female	304	64.1
Age	≦18	3	0.6
	18–25	467	98.5
	≧26	4	0.9
Subject	Science and Engineering	230	48.5
	Social science	244	51.5
education	Junior college students	105	22.2
	Undergraduate	310	65.4
	Graduate student	59	12.4
Social media usage history (month)	≦6	6	1.3
	6–12	6	1.3
	13–24	17	3.5
	25–36	35	7.4
	≧36	410	86.5
Number of online friends (person)	≦100	61	12.9
	101–200	148	31.2
	201–300	133	28.1
	301–400	39	8.2
	≧400	93	19.6
Social media usage duration (hours)	≦1	7	1.5
	1–2	86	18.1
	2–3	68	14.3
	3–4	97	20.5
	≦4	216	45.6

### Analysis of sample reliability and validity

#### Factor analysis and standard method bias analysis

The results of the exploratory factor analysis indicate that the coefficient of the KMO test is 0.928, and the *p* value in Bartlett’s test of sphericity is less than 0.001, suggesting that it is suitable for factor analysis. Five factors with eigenvalues greater than one were obtained by rotating the factors using principal component and maximum variance methods. The eigenvalue of the sixth factor (0.959) is also close to 1. The cumulative variance contribution rate of factor analysis is 64.160%, indicating that the entire questionnaire achieves an excellent level. In this study, the explanatory power of the first factor is 30.059%, which is lower than the standard method bias threshold of 50% (Podsakoff et al., 2003). Therefore, this study’s data has no standard method bias issue.

#### Reliability and validity testing of variables

According to the analysis results in Table 2, it can be seen that the factor loadings of each scale are all greater than 0.5, indicating good convergent validity. Cronbach’s alpha coefficients range from 0.811 to 0.919, which is an ideal state. Furthermore, among the variables, except for the AVE values of the social media use and Subjective well-being scales being slightly below the standard value of 0.5, the other four scales meet the requirements well. The CR values are generally above 0.8, indicating that the measured variables consistently explain the latent variable. In summary, each scale has good reliability and validity.

**Table 2:** Results of variable reliability and validity testing.

Variable	Items	Factor loadings	Cronbach’s alpha	AVE	CR
Social media use	SM1	0.642	0.811	0.432	0.818
	SM2	0.624			
	SM3	0.758			
	SM4	0.763			
	SM5	0.574			
	SM6	0.553			
Bridging social capital	BR1	0.659	0.881	0.528	0.886
	BR2	0.801			
	BR3	0.762			
	BR5	0.696			
	BR6	0.703			
	BR7	0.811			
	BR9	0.635			
Bonding social capital	BO1	0.789	0.871	0.587	0.876
	BO2	0.655			
	BO3	0.802			
	BO4	0.749			
	BO5	0.825			
Maintaining social capital	MA1	0.912	0.919	0.697	0.920
	MA2	0.869			
	MA3	0.797			
	MA4	0.788			
	MA5	0.802			
College attachment	CA1	0.755	0.826	0.665	0.856
	CA2	0.860			
	CA3	0.828			
Subjective well-being	WB1	0.496	0.884	0.437	0.792
	WB2	0.647			
	WB3	0.772			
	WB4	0.662			
	WB5	0.697			

AVE, Average Variance Extracted; CR, Composite Reliability.

#### Correlation analysis and discriminant validity

According to Table 3, the mean of each variable on the 7-point scale is greater than 4, indicating that the respondents hold a positive attitude or evaluation toward these variables. Social media use is not significantly correlated with bridging social capital, but it is significantly correlated with other variables. All other variables significantly correlate with subjective well-being except for maintaining social capital. Moreover, maintaining social capital is only significantly correlated with social media use and college attachment. In the discriminant validity test, the standardized correlation coefficients between each pair of dimensions are all less than the square root of the AVE value corresponding to the dimension, thus indicating that there is good discriminant validity among all dimensions.

**Table 3:** Correlation matrix and discriminant validity test.

Variable	M	SD	SM	BR	BO	MA	CA	WB
SM	4.68	1.19	**0.646**					
BR	4.19	0.96	0.084	**0.886**				
BO	4.35	1.02	0.101*	0.779***	**0.876**			
MA	4.49	1.40	0.477***	−0.052	−0.044	**0.920**		
CA	4.45	1.14	0.232***	−0.194***	−0.160***	0.416***	**0.815**	
WB	4.64	0.98	0.139**	0.772***	0.793***	−0.033	−0.125**	**0.792**

*p < 0.05, **p < 0.01, ***p < 0.001, SM, Social Media use; BR, Bridging social capital; BO, Bonding social capital; MA, Maintaining Social Capital; CA, College attachment; WB, Subjective well-being. The bolded values represent the square roots of AVE.

### Difference test

In this analysis, independent sample t-tests and one-way ANOVA were primarily utilized to investigate the impact of differences in various dimensions of variables on subjective well-being based on the characteristics under consideration. According to the t-test results, there were no significant gender differences in the performance of male and female international students across all dimensions. Similarly, the one-way ANOVA results indicated no notable disparities based on age, nationality, discipline, educational background, social media use history, number of friends, or usage duration.

### Test of fit for structural equation model

**Figure 2:**
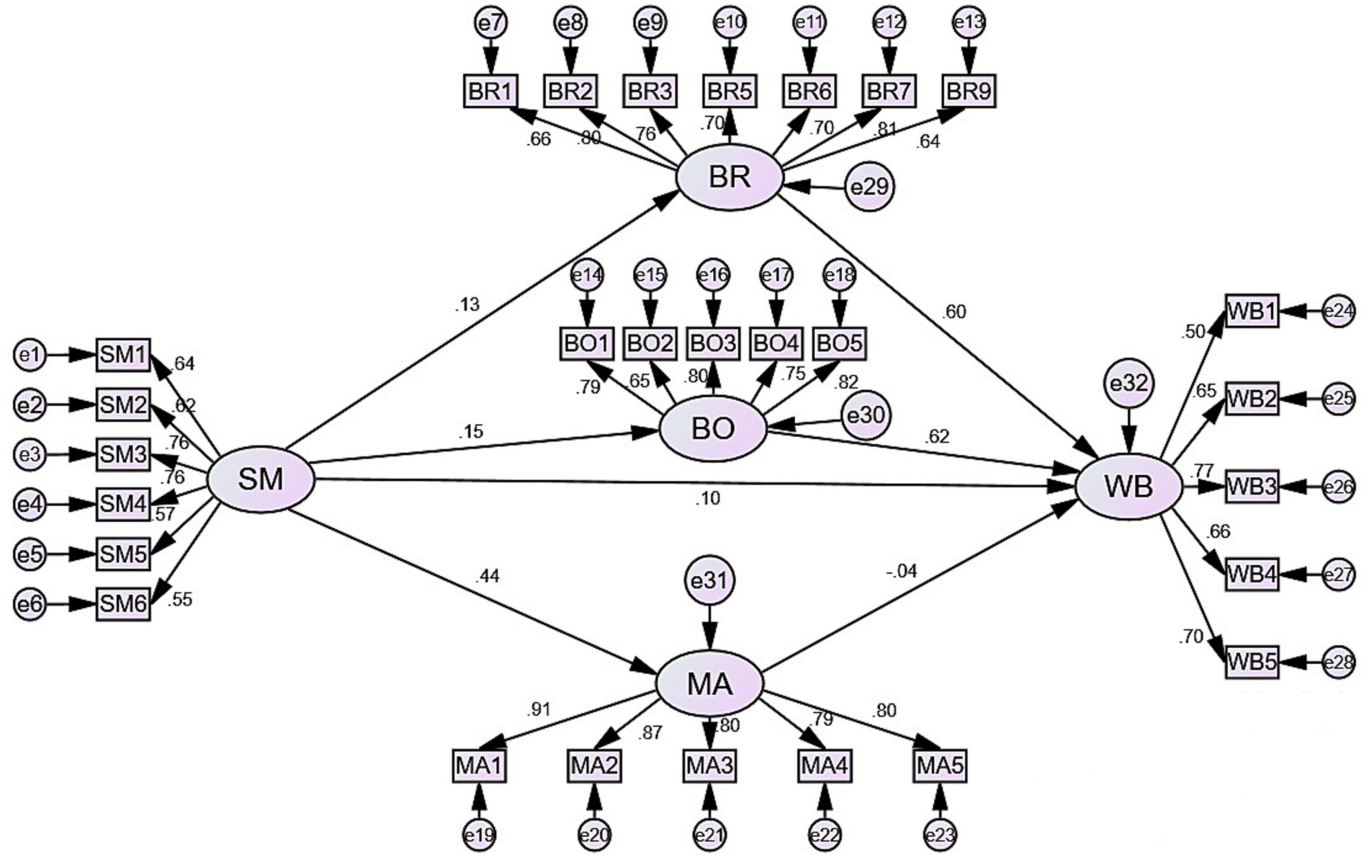
SEM measurement model result.

Figure 2 presents the results of the structural equation modeling. The CMIN/DF ratio is 3.889, falling within the range of 3–5. The RMSEA value is 0.078, within the acceptable range of less than 0.08. Additionally, the test results for GFI, IFI, and CFI all meet the adequate level of above 0.8 (Fornell and Larcker, 1981). Therefore, the measurement model for the relationship between social media use and subjective well-being exhibits a good fit.

### Direct effect path analysis

According to the analysis results in Table 4, it can be seen that in this path hypothesis test, social media use significantly and positively predicts subjective well-being (*β* = 0.104, *p* < 0.05). Social media use significantly and positively predicts bridging social capital (*β* = 0.126, *p* < 0.05), bonding social capital (*β* = 0.148, *p* < 0.01), and maintaining social capital (*β* = 0.435, *p* < 0.001). Bridging social capital significantly and positively predicts subjective well-being (*β* = 0.605, *p* < 0.001), and bonding social capital significantly and positively predicts subjective well-being (*β* = 0.625, *p* < 0.001). However, maintaining social capital does not have a regression relationship with subjective well-being (*β* = −0.037, *p* > 0.05). Therefore, H1–H6 is supported, while H7 is not supported.

**Table 4:** Results of path coefficients in structural equation modeling.

Path			Estimate	S.E.	C.R.	*p*
WB	←	SM	0.104	0.022	2.380	0.017*
BR	←	SM	0.126	0.041	2.344	0.019*
BO	←	SM	0.148	0.045	2.747	0.006**
MA	←	SM	0.435	0.066	7.958	0.000***
WB	←	BR	0.605	0.048	8.557	0.000***
WB	←	BO	0.625	0.041	9.326	0.000***
WB	←	MA	−0.037	0.017	−0.941	0.346

S.E., Standard Error; C.R., Critical Ratio. *p < 0.05, **p < 0.01, ***p < 0.001; SM, Social Media use; BR, Bridging social capital; BO, Bonding social capital; MA, Maintaining Social Capital; CA, College attachment; WB, Subjective well-being.

### Test of the mediating effect of social capital

Social capital, as a mediating variable, has three dimensions. We will specifically analyze the mediating role of each dimension to determine whether it is significant. Since maintaining Social Capital does not significantly impact the path relationship with subjective well-being, we will further analyze the mediating effects of bridging social capital and bonding social capital. We employ the method of “Indirect effect of x on Y through Mi = a_i_b_i_, the direct effect of X on Y = c’” (Wen et al., 2022) and utilize Bootstrap technology to test the mediating role of these two types of social capital in the relationship between social media use and subjective well-being.

Testing the mediating effect of bridging social capital. In the path model of social media use on subjective well-being, the mediating effect of bridging social capital is significant in terms of both the direct impact (*β* = 0.054, *p* < 0.05) and the total effect (*β* = 0.093, *p* < 0.01). In contrast, the indirect impact (*β* = 0.039, *p* > 0.05) is insignificant. Therefore, bridging social capital has no mediating effect on this path. Specific data are shown in Table 5.

**Table 5:** Test results of the mediating effect of bridging social capital.

Parameter	Estimate	LLCI	ULCI	*p*	Percentage
Indirect effects	0.039	−0.001	0.081	0.057	41.9%
Direct effect	0.054	0.011	0.097	0.012^*^	58.1%
Total effect	0.093	0.039	0.150	0.003^**^	

LLCI, Lower Limit of Confidence Interval; ULCI, Upper Limit of Confidence Interval. *p < 0.05, **p < 0.01.

Testing the mediating effect of bonding social capital. As can be seen from Table 6, in the path model of social media use on subjective well-being, the direct effect (*β* = 0.054, *p* < 0.05), indirect effect (*β* = 0.048, *p* < 0.05), and total effect (*β* = 0.101, *p* < 0.01) of the mediating effect of bonding social capital are all significant. Therefore, bonding social capital plays a partial mediating role in this path.

**Table 6:** Test results of the mediating effect of bonding social capital.

Parameter	Estimate	LLCI	ULCI	*p*	Percentage
Indirect effects	0.048	0.005	0.096	0.024^*^	47.5%
Direct effect	0.054	0.011	0.097	0.012^*^	52.5%
Total effect	0.101	0.046	0.161	0.001^**^	

LLCI, Lower Limit of Confidence Interval; ULCI, Upper Limit of Confidence Interval. *p < 0.05, **p < 0.01.

In summary, the results of the mediation effect test for social capital indicate that H9 is supported, while H8 and H10 are not. That is to say, only the mediation effect of bonding social capital is significant among the three dimensions of social capital.

### Test of moderating effect

On the premise that the direct effect holds, this study utilized the eighth model of the SPSS plugin PCOCESS4.1 (Hayes, 2013) to examine whether college attachment plays a moderating role in the relationship between social media use and subjective well-being, acting as a mediator (bridging social capital). Initially, we processed the independent and moderating variables by plus or minus one standard deviation, forming two groups with different levels of social media use and college attachment. The test results are presented in Table 7. In the direct impact relationship between social media use and subjective well-being, the interaction term between social media use and college attachment (*β* = 0.000, *t* = −0.018, *p* = 0.985) has a 95% confidence interval of [−0.040, 0.040], which includes 0, indicating that the moderating effect is not significant. Therefore, H11 is not supported. In the indirect impact relationship between social media use and subjective well-being, the interaction term between social media use and college attachment (*β* = 0.070, *t* = 2.329, *p* = 0.020) has a 95% confidence interval of [0.011, 0.130], which does not include 0, indicating that the moderating effect is significant. Hence, H12 is supported. This suggests that college attachment only moderates the relationship between social media use and subjective well-being, specifically within a mediated moderating effect.

**Table 7:** Results of the moderating effect test of college attachment.

Predictors	Model 1 (BR)		Model 2 (WB)	
	*β*	t	*β*	t
SM	0.118	3.182^**^	0.060	2.367^*^
CA	−0.199	−5.138^***^	0.007	0.243^*^
BR			0.788	25.442^***^
SM*CA	0.070	2.329^*^	0.000	−0.018
*R^2^*	0.066		0.602	
*F*	11.123		176.999	

*p < 0.05, **p < 0.01, ***p < 0.001, SM, Social Media use; BR, Bridging social capital; MA, Maintaining Social Capital; CA, College attachment; WB, Subjective well-being.

**Figure 3:**
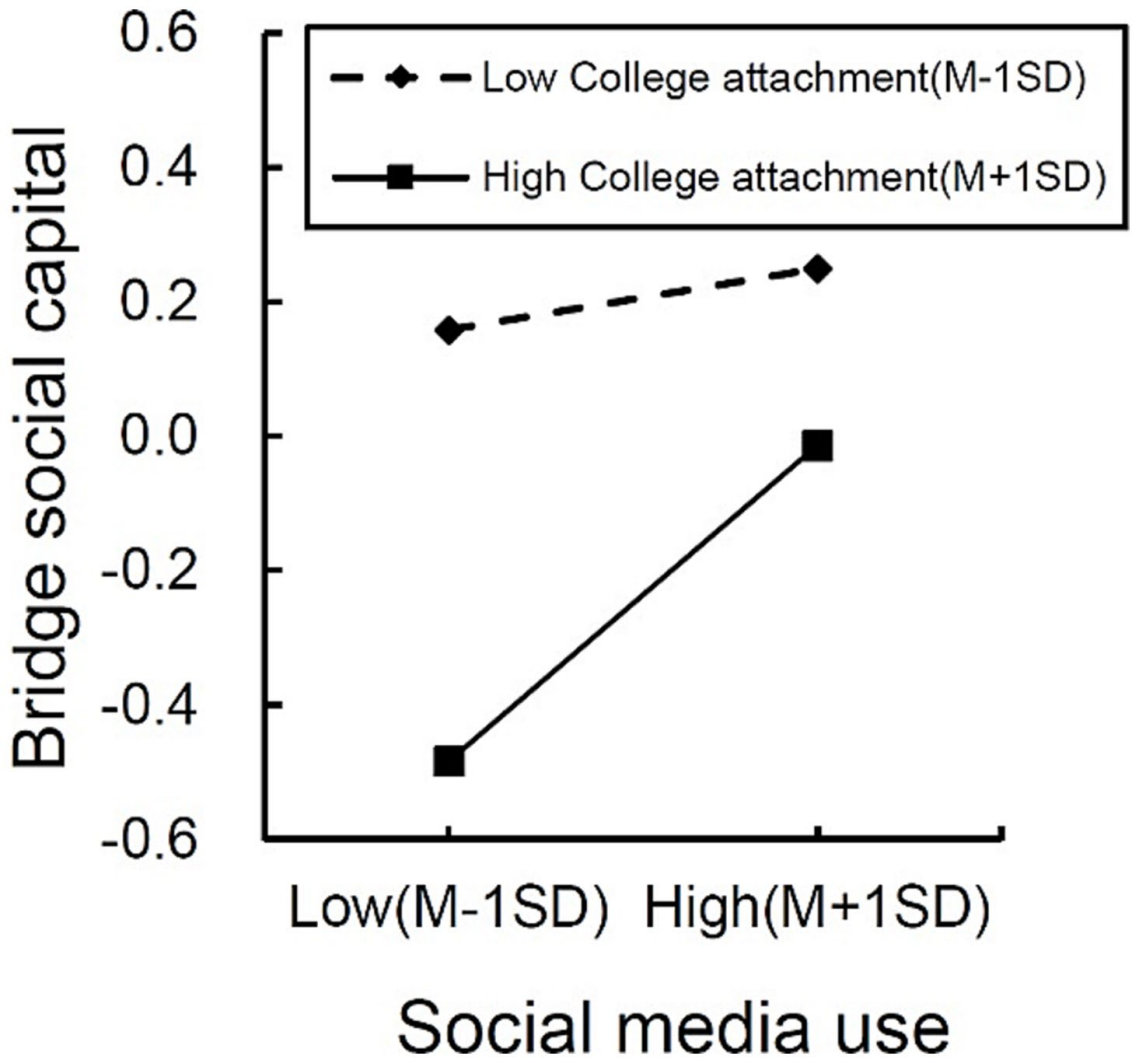
Moderating role of college attachment.

To better explain the moderating effect of college attachment, we used a simple slope to examine Figure 3, which illustrates the relationship between social media use and subjective well-being among two college attachment groups. Simple slope tests showed that for international students with low college attachment, social media use did not significantly predict bridging social capital (*β*_simple_ = 0.038, *p* > 0.05). However, for international students with high college attachment, social media use significantly predicted bridging social capital (*β*_simple_ = 0.198, *p* < 0.001). In the high college attachment group (M + 1SD), individuals’ subjective well-being increases as the degree of social media use increases, indicating that college attachment plays a positive moderating role. In other words, when international students have a higher level of college attachment, their use of social media will significantly impact their subjective well-being.

## Discussion

This study first systematically explored the mediating mechanism of social capital in the impact of social media use on the subjective well-being of international students and revealed the moderating role of college attachment. The results show that social media use directly enhances international students’ subjective well-being and indirectly does so through bonding social capital. Unexpectedly, the mediating effects of bridging and maintaining social capital were insignificant, while college attachment positively moderated the relationship between social media use and bridging social capital. Overall, international students use social media to establish groups to seek various needs in learning and life. This effectively reduces personal loneliness and expands their friend network, improving life satisfaction. Therefore, although social media use has many negative impacts on most people, it is an important tool for international students to gain subjective well-being. These findings offer a new perspective on understanding the psychological mechanisms of cross-cultural adaptation in the digital age.

Consistent with prior studies (Gao, 2022; Hu and Guo, 2021; Koc and Turan, 2021; Lin et al., 2022), social media use positively predicted subjective well-being. The more international students engage with different types of friends on social media, the more social support they receive and the higher their subjective well-being. Notably, using social media to form groups for academic and living needs reduces loneliness and expands social networks, thereby improving life satisfaction. Despite the potential adverse effects of social media on most populations, it serves as a crucial tool for enhancing the subjective well-being of international students. Some international students only live in a comfortable communication circle with their compatriots or other international friends using social media, reducing or not having online or offline communication with friends in the host country (Yang, 2018). There is a high risk of dependence on social media, which is not conducive to acquiring subjective well-being. Relevant individuals need to understand that social media is only a means of support for connecting with people in daily life rather than an end in itself.

This study confirms the applicability of online social capital theory in digital cross-cultural adaptation. Bonding social capital emerged as the core mediator between social media use and well-being, supporting the dominance of “strong ties” in psychological well-being, which aligns with Reimann et al. (2023). Similar to prior studies (Kuang and Wu, 2019; Lee and Cho, 2018; Wang et al., 2018; Xu et al., 2021), social media use significantly predicted social capital, which in turn predicted subjective well-being (Hu and Chen, 2017; Pang, 2018c). Among the three types of social capital, only bonding social capital mediated the relationship between social media use and subjective well-being, consistent with Lin et al. (2022) and Chen and Li (2017). This suggests that new friendships international students form can bridge the gap to enhance subjective well-being in a foreign context (Du and Lin, 2019). International students with low life satisfaction tend to seek online social networks to improve their happiness, and those active on social media report feeling happier. This indicates a positive correlation between social capital, personal well-being, and quality of life. However, over-reliance on social media to connect with home country relatives and friends at the expense of face-to-face interactions with host country peers may lead to social media dependency, which is detrimental to well-being.

The moderating effect of bridging social capital was only significant at high levels of college attachment, likely because highly attached individuals more actively convert weak online ties into strong offline connections (Ellison et al., 2014). Maintaining social capital did not significantly predict subjective well-being, contradicting Pang (2018a), possibly because it primarily relates to the home country’s social networks. In contrast, the subject well-being depends more on the newly established host country’s social support (Billedo et al., 2019). According to the double-edged sword effect of social capital (Erfani and Abedin, 2018), over-reliance on home country social networks may hinder cultural integration and offset psychological benefits, consistent with Billedo et al. (2019), who found better adaptation among those with more host country interactions and vice versa. Thus, maintaining capital may depend on balancing usage intensity with cultural engagement, suggesting future research should use longitudinal designs to explore its dynamic effects.

Importantly, this study found that college attachment moderates the relationship between social media use and bridging social capital, a finding not previously documented. For international students with low university attachment, their higher bridging social capital may indicate a tendency to seek support and resources through extensive weak-tie networks when adapting to a new environment. This could be a compensatory mechanism for the lack of belonging to the university. Bridging social capital provides them with more information and opportunities, aiding their integration into the host society. However, high reliance on bridging social capital may also suggest difficulties forming deep and trusting relationships, affecting their long-term adaptation and mental health. Thus, while bridging social capital is beneficial in the short term, they may need more bonding social capital for sustained mental well-being and happiness. Thus, the moderating role of college attachment is critical and deserves stakeholders’ attention.

In summary, this study extends existing theories in three ways: First, it confirms the ‘resource gain’ path of social media use in cross-cultural adaptation (Hobfoll, 1989), highlighting the mediating advantage of bonding social capital. Second, it identifies college attachment as a moderator, refining the boundary conditions of the online social capital theory. Third, it reveals the complex role of maintaining capital, challenging the universality of traditional social capital classifications in cross-cultural contexts and suggesting the need for context-specific theoretical frameworks.

## Conclusion and suggestions

International students often face significant social conflicts in their host countries due to language and cultural differences. In the process of cross-cultural adaptation, social media has become an essential tool for international students’ lives and studies, and it is a significant factor affecting their subjective well-being. This study finds that social media use indirectly impacts international students’ subjective well-being through the mediating effect of bonding social capital. Furthermore, the mediating role of social capital is also moderated by college attachment. Previous studies have only emphasized the direct effect of social media use on subjective well-being, neglecting the indirect impact of social capital and college attachment. This study suggests that administrators can focus on enhancing international students’ sense of belonging to the university in their daily management and actively utilize social media to guide students to participate in meaningful social media groups, thereby improving their subjective well-being. On the one hand, students can be guided to use social media reasonably to prevent excessive dependence on friends from their home country, which may lead to failure in cross-cultural adaptation. On the other hand, administrators can organize various group activities to increase opportunities for international students to interact with other students, reduce the likelihood of loneliness, and enhance subjective well-being. Additionally, administrators should improve universities’ hardware and software levels to strengthen international students’ recognition of the university and gain a sense of belonging, further promoting the generation of happiness.

While this study has yielded novel insights, future research should address several limitations. First, the cross-sectional design precludes inferences about causal relationships. Future studies could employ the Experience Sampling Method (ESM) to track dynamic processes over time. Second, the sample was drawn from universities in Guangxi, which may limit generalizability due to regional developmental specificities. National or cross-country comparative studies are recommended to enhance the applicability of the findings. Third, this study did not differentiate between types of social media (e.g., those oriented toward strong vs. weak ties or active vs. passive use), which may influence outcomes differently. For future research, we suggest (1) incorporating physiological markers (e.g., cortisol levels) to assess well-being multimodally; (2) investigating how algorithmic recommendations shape social capital structures among international students; and (3) developing a “cultural adaptation–social media use” matching model to inform personalized interventions.

## Data Availability

The original contributions presented in the study are included in the article/supplementary material, further inquiries can be directed to the corresponding author.
